# Cefiderocol for Severe Carbapenem-Resistant *A. baumannii* Pneumonia: Towards the Comprehension of Its Place in Therapy

**DOI:** 10.3390/antibiotics11010003

**Published:** 2021-12-21

**Authors:** Emanuele Rando, Francesco Vladimiro Segala, Joel Vargas, Cristina Seguiti, Gennaro De Pascale, Rita Murri, Massimo Fantoni

**Affiliations:** 1 Clinic of Infectious Diseases, Catholic University of the Sacred Heart, 00168 Rome, Italy; francescovladimiro.segala@policlinicogemelli.it (F.V.S.); cristina.seguiti@policlinicogemelli.it (C.S.); rita.murri@policlinicogemelli.it (R.M.); massimo.fantoni@policlinicogemelli.it (M.F.); 2 Department of Laboratory and Infectious Diseases Sciences, Fondazione Policlinico Universitario Agostino Gemelli IRCCS, 00168 Rome, Italy; 3 Department of Emergency, Anesthesiological and Reanimation Sciences, Fondazione Policlinico Universitario Agostino Gemelli IRCCS, 00168 Rome, Italy; joel.vargas@policlinicogemelli.it (J.V.); gennaro.depascale@policlinicogemelli.it (G.D.P.)

**Keywords:** cefiderocol, pneumonia, *A. baumannii*, CRAB

## Abstract

Cefiderocol use in *A. baumannii* pneumonia still represents an important matter of debate. The aim of this study is to describe 13 cases of carbapenem-resistant *A. baumannii* (CRAB) pneumonia treated with cefiderocol in real-life practice. We retrospectively included patients with CRAB pneumonia hospitalized at Fondazione Policlinico Universitario Agostino Gemelli Hospital treated with cefiderocol either in the general ward or the intensive care unit. A total of 11 patients out of 13 had ventilator-associated pneumonia caused by CRAB, and 12/13 patients had polymicrobial infection. We found a 30-day success rate of 54%. Cefiderocol may have a role when facing severe XDR *A. baumannii* pneumonia. Future studies are warranted to better define its place in therapy in CRAB infections.

## 1. Introduction 

Carbapenem-resistant Gram-negative bacteria (GNB) continue to represent a major public health issue worldwide [1], posing an increasing burden in terms of morbidity and mortality in several areas of the world [2] and, in Europe, Italy ranks among the most affected countries [3]. Despite the global concern over extended spectrum beta-lactamase (ESBL) and carbapenemase-producing *Enterobacterales*, difficult to treat infections due to non-fermenting GNB are being increasingly reported worldwide [4]. Among them, carbapenem-resistant *A. baumannii* (CRAB) poses a particular risk, being classified as an “urgent threat” by the Centers of Disease Control and Prevention [5] due to its extremely limited treatment options [6] and its capacity to survive on healthcare facility surfaces and shared medical equipment [7]. Furthermore, healthcare disruption due to the COVID-19 pandemic, especially within intensive care units (ICUs), may have led to an increase in CRAB nosocomial outbreaks [8]. In particular, a recent metanalysis [9] showed that nearly half of critically ill COVID-19 patients admitted into the ICU develop ventilator-associated pneumonia, with mortality rates as high as 42%.

Among novel antibiotics, cefiderocol, a siderophore cephalosporin, has demonstrated in vitro and in vivo activity against a broad range of Gram-negative bacteria [10,11]. Despite these promising results, a limited number of studies has been conducted so far, and its place in therapy is not well-established yet, especially in ventilator-associated pneumonia [12] and polymicrobial infections. In this case series, we aim to illustrate our experience with cefiderocol in patients with HAV/VAP due to carbapenem-resistant *A*. *baumannii.*

## 2. Materials and Methods

A retrospective study of patients with HAP or VAP caused by at least one MDR Gram-negative bacteria treated with cefiderocol either in an ordinary ward or the ICU of Policlinico Agostino Gemelli Hospital from 1 January 2021 to 15 November 2021 was performed. Data from all participants were anonymously collected in an electronic dataset.

Cefiderocol was prescribed at the discretion of an infectious diseases or critical care specialist if clinically indicated based on patient status and microbiological isolates, both as a first-line and second-line treatment. The administration of cefiderocol consisted of an extended infusion of 3 h adjusted for renal function either in 100 mL or 250 mL of normal saline, following productor indication.

Clinical cure was defined as the resolution of signs and symptoms, resolving respiratory failure and clinical/laboratory evidence of improvement. Data on survival status were recorded at 7, 14 and 30 days from the diagnosis of pneumonia. Adverse events attributable to cefiderocol use were also recorded. Monotherapy and combination therapy were defined as cefiderocol use only as an intravenous anti-Gram-negative agent or with other active agents, respectively.

Continuous variables were described using median and interquartile ranges, and categorical variables were described using frequencies and percentages.

## 3. Results

A total of 14 patients treated with cefiderocol for severe, polymicrobial pneumonia were included in the study (Table 1). All patients were male, and the mean age (interquartile range; IQR) was 61 (IQR, 52–66) years. Overall, 12 out of 13 patients (92.3%) were hospitalized for COVID-19, of whom 83.3% (10/12) developed severe respiratory failure and were then admitted into ICU. Among the two patients hospitalized for reasons not related to COVID-19, one had idiopathic pulmonary fibrosis, and the other suffered from recurrent bacterial pneumonia.

A total of 10 patients (76.9%) developed ventilator-associated pneumonia. In all included subjects, *A. baumannii* was identified among the pathogens. All the isolates were resistant to all tested antimicrobials except colistin. It is notable that 12/13 (92.3%) infections were polymicrobial. Along with *A. baumannii,* the other isolated pathogens included *P. aeruginosa* (46.2%), *S. aureus* (53.8%), *K. pneumoniae* (38.5%), and *E. coli* (15.4%). Among *P. aeruginosa* and *K. pneumoniae* isolates, respectively, 33.3% (2/6) and 40% (2/5) were resistant to carbapenems. Conversely, 6/13 (46.1%) *S. aureus* specimens were MRSA.

In our population, cefiderocol was prescribed as part of the first-line therapy in 30.7% (4/13) of patients and administered as a monotherapy in 61.5% (8/13) of cases. The median duration of cefiderocol treatment was 10 (IQR, 7–12.5) days. When cefiderocol was prescribed as part of the second-line therapy (9/13, 69.2%), patients were experiencing a clinical failure of a previous colistin-based regimen in 88.9% (8/9) of cases. Clinical cure was achieved in 7 subjects, while failures were all due to death (6/13, 46.2%). For patients who did not survive, the main causes of death were respiratory failure (3/6, 50%) and septic shock (3/6, 50%). In this study, no major adverse events due to the cefiderocol were recorded.

## 4. Discussion

In this study, we reported our real-life experience with cefiderocol when facing carbapenem-resistant *A. baumannii* as the causative pathogen in pneumonia both in general wards and the ICU. Of note, a 30-day success rate of 54% was found. 

HAP and VAP are lethal conditions frequently encountered in clinical practice, and they account for a high rate of mortality [13,14], especially when caused by carbapenem-resistant *A. baumannii* with limited therapeutic options [15]. In this case, cefiderocol may represent an important tool for clinicians in treating extensively resistant Gram-negative bacteria [16], displaying an interesting profile of intrapulmonary penetration [17] and permitting the unfavorable phenomena of toxicity of colistin to be avoided [18]. Despite these premises, cefiderocol effectiveness in CRAB infections, especially pneumonia, has been questioned by the CREDIBLE-CR study. However, subsequent published reports drew considerable attention to the utility and efficacy of this molecule when confronting *A. baumannii* infections [19,20,21,22]. 

In this real-life experience, cefiderocol represented an effective treatment for 7 of 13 cases. These results are noticeable since only patients with HAP/VAP by CRAB were included; the majority of patients were severely ill and with respiratory deterioration prior to pneumonia onset. Indeed, 11 of 13 cases were treated in ICU, and 11 had critical SARS-CoV-2 pneumonia requiring mechanical ventilation and, in 2 cases, extracorporeal membrane oxygenation. In all cases except one, patients had severe polymicrobial pneumonia with multiple Gram-negative isolates, including CRAB, XDR *P. aeruginosa* and KPC-producer *K. pneumoniae*, among others. We found a 30-day mortality of 46%, consistent with previous studies exploring XDR *A. baumannii* pneumonia [23,24] and VAP in SARS-CoV-2 infection [9]. This is remarkable considering the complexity of patients treated in the critical care setting and the safety profile of this molecule [25]. ARDS and septic shock were the main causes of death in our cohort, probably reflecting SARS-CoV-2-related respiratory failure. The relationship between COVID-19 and *A. baumannii* remains intimate; CRAB is a well-known in-hospital colonizer, and its pathogenic role is especially evident in vulnerable individuals, including ICU and ventilated patients. As a result of the pandemic, a considerable number of patients have been mechanically ventilated in ICUs, contributing to the spread of this bacterium [26]. Despite the frailty of patients infected by *A. baumannii*, CRAB infection has been associated with an increased risk of mortality [27], even when occurring in COVID-19 patients [28]. This may be related to the interdependence between CRAB infection and severely ill patients along with the unpredictable pharmacokinetic/pharmacodynamic profile of colistin, which is still the most-used drug for CRAB pneumonia. In fact, colistin has been associated with a low concentration in the epithelial lining fluid [29], which is particularly inconvenient when treating critical patients. Considering these facts, a novel and more predictable treatment for this kind of infection appears particularly crucial.

Moreover, cefiderocol was used as monotherapy in more than half of patients; the choice of monotherapy with respect to combination reflects, on one hand, a prescription of the drug as “rescue”, in response to the clinical failure of previous antimicrobial regimens. On the other hand, it is the result of clinical judgement in light of patient presentation, laboratory profile, antibiotic toxicity—with special attention to colistin nephrotoxicity—and available evidence at the time the study drug was prescribed. For instance, a monotherapy was preferred when hemodynamic instability was not an immediate concern and cefiderocol susceptibility testing was available, whereas combination therapy was reserved for patients requiring vasopressors or with severe deterioration of respiratory status. With this in mind, severely ill patients could have been more represented in the combination therapy group compared to the monotherapy group. Indeed, a patient initially treated with meropenem for carbapenem-susceptible *P. aeruginosa* VAP relapsed after 7 days of treatment, becoming febrile and eventually developing sepsis. A new sample from tracheal aspirate was obtained, and CRAB and carbapenem-resistant *P. aeruginosa* were isolated. In this case, considering the hemodynamic stability and bacterial susceptibility profiles, a 14-course of cefiderocol monotherapy was started with complete resolution of pneumonia. However, the high rate of mortality seen in the monotherapy group is also due to the use of cefiderocol as a last resort, even in critical patients when prior treatment failed to reach clinical improvement along with substantial drug toxicity.

Nevertheless, our study should be interpreted considering several limitations. First, it is a case series from a single center and has the shortcomings of this kind of study. Second, due to the limited number of patients included in this cohort and the descriptive nature of this study, no definitive conclusions can be drawn about the efficacy of cefiderocol. Third, we did not include microbiological outcomes because, given the retrospective nature of the study, it was not available for all patients. Fourth, due to the prevalence of severe COVID-19 patients, it is difficult to establish the actual mortality of HAP/VAP due to CRAB. Fifth, many cases included in this case series were polymicrobial infections, possibly altering the contribution of *A. baumannii* in defining the severity of the disease, especially when *P. aeruginosa* or *S. aureus* were also present. Despite the aforementioned limitations, our work may contribute to redefining cefiderocol’s place in therapy in treating severe carbapenem-resistant *A. baumannii* pneumonia, adding new information on clinical experience with this drug. These results are particularly important as a consequence of the considerable debate about cefiderocol use in lower respiratory tract infections. However, further well-designed studies are needed to clarify cefiderocol indications in clinical practice.

## Figures and Tables

**Table 1:** Description of the 13 patients who developed HAP/VAP due to *A. baumannii* treated with cefiderocol.

Age/Sex	Underlying Diseases	ICU Admission, Reason	Type of Pneumonia	Isolated Pathogens	Treatment Regimens ^a^	CFD Dosage and Duration (Days)	Monotherapy ^b^	30-Day Outcome
M/63	COVID-19, Parkinson disease	COVID-19	VAP	CRAB CRPA	MEM/COL + CAZ-AVI/CFD	2 g q6h; 12 d	Yes	Clinical cure
M/62	COVID-19, COPD, obesity, DM type II, HBP	COVID-19	HAP	CRAB *P. aeruginosa*	COL + CAZ-AVI/CFD	2 g q8h; 7 d	Yes	Clinical cure
M/74	COVID-19, DM type II, HBP	Not admitted into ICU	HAP	CRAB CRPA	CFD + aerosolized COL + ISZ	2 g q8h; 18 d	Yes	Clinical cure
M/66	COVID-19, HBP, meningioma	Not admitted into ICU	HAP	CRAB *P. aeruginosa* *E. coli* XDR *K. pneumoniae* *P. mirabilis* MRSA	MEM + TP + COL/CFD + TP	2 g q8h; 7 d	Yes	Clinical cure
M/65	COVID-19, DM type II	COVID-19	VAP	CRAB MRSA	TGC + COL + RMP + LZD/CFD + aerosolized COL + LZD	1 g q6h; 10 d	Yes	Clinical cure
M/56	COVID-19, IPF, DM type II, AML, GVHD	COVID-19	HAP	CRAB *S. marcescens* *P. aeruginosa* *K. pneumoniae*	CFD + COL/FEP + CIP	2 g q8h; 4 d	No	Clinical cure
M/52	COVID-19	COVID-19	VAP	CRAB *K. pneumoniae* MSSA	AXO + OXA/CFD + aerosolized COL + OXA + FO/CFD + OXA + COL + MTZ + CAS	2 g q8h; 27 d	No	Death
M/79	COVID-19, COPD, HBP, PAD	COVID-19	VAP	CRAB *E. coli* CTX-M + MRSA	CFD + VAN + aerosolized COL	2 g q8h; 10 d	Yes	Death
M/58	COVID-19	COVID-19	VAP	CRAB *K. pneumoniae* *P. aeruginosa* MRSA	AMC/IMI + COL + LNZ/CFD	2 g q8h; 10 d	Yes	Clinical cure
M/66	COVID-19, HBP	COVID-19	VAP	CRAB *E. cloacae* MRSA	MEM + TGC + COL/CFD + TGC + aerosolized COL + ABLC	2 g q8h; 4 d	No	Death
M/39	Cognitive impairment, obesity, recurrent pneumonia	Pneumonia	VAP	CRAB *K. pneumoniae* KPC MRSA	COL + TGC + FO/CFD + aerosolozed COL	2 g q8h; 10 d	Yes	Death
M/35	COVID-19	COVID-19	VAP	CRAB *K. pneumoniae* KPC	CAZ-AVI + COL + OXA/CFD + FO	2 g q8h; 9 d	No	Death
M/42	COVID-19	COVID-19	VAP	CRAB	CFD + COL + FO	2 g q8h; 13 d	No	Death

HAP: hospital-acquired pneumonia; VAP: ventilator-associated pneumonia; DM: diabetes mellitus; HBP: high blood pressure, COPD: chronic obstructive pulmonary disease; IPF: idiopathic pulmonary fibrosis; AML: acute myeloid leukemia; GVHD: graft versus host disease; PAD: peripheral artery disease; CRAB: carbapenem-resistant *A. baumannii*; CRPA: carbapenem-resistant *P. aeruginosa*; MRSA: methicillin-resistant *S. aureus;* MSSA: methicillin-susceptible *S. aureus*; XDR: extensively drug resistant; CFD: cefiderocol; MEM: meropenem; COL: colistin; CAZ-AVI: ceftazidime-avibactam; ISZ: isavuconazol; TP: teicoplanin; TGC: tigecycline; RMP: rifampicin; LZD: linezolid; FEP: cefepime; CIP: ciprofloxacin; AXO: ceftriaxone; OXA: oxacillin; FO: fosfomycin; MTZ: metronidazole; CAS: caspofungin; AMC: amoxicillin/clavulanic acid; IMI: imipenem; ABLC: amphotericin B lipid complex. ^a^ The symbol/indicates temporally consecutive regimens. ^b^ Monotherapy is defined as cefiderocol use as the single intravenous anti-Gram-negative agent.

## Data Availability

All data relevant to the study is included in the article and is available from the corresponding author upon request.
